# A Not-So-Good Way to Die? Respiratory Syncytial Virus–induced Necroptotic Cell Death Promotes Inflammation and Type 2–mediated Pathology

**DOI:** 10.1164/rccm.202003-0533ED

**Published:** 2020-06-01

**Authors:** James A. Harker, Robert J. Snelgrove

**Affiliations:** ^1^ National Heart and Lung Institute Imperial College London London, United Kingdom

Respiratory syncytial virus (RSV) is a significant cause of acute respiratory infection in infants, and most children have been infected at least once before the age of 2 years (1). A small percentage of these children develop viral bronchiolitis and pneumonia associated with intense inflammation in their lower airways. Accordingly, RSV is one of the most significant causes of infant hospitalization in the developed world and a significant cause of infant mortality in the developing world. RSV-dependent lower respiratory tract infections in early life are also associated with an increased prevalence of wheeze and asthma in later life. Despite over 60 years of research into RSV, there is currently no licensed vaccine. In addition, although prophylactic administration of a monoclonal antibody against the RSV F protein can successfully prevent RSV bronchiolitis, its administration after infection has limited benefit. What drives severe disease during RSV-dependent lower respiratory tract infections and which pathways might be therapeutically targeted after infection therefore remain significant questions.

Airway epithelial cell (AEC) death is prevalent with respiratory viral infection, but the type of death elicited can profoundly impact host immunity and ensuing pathology. Apoptosis is an ordered, noninflammatory cell death that is an efficient method of removing virally infected cells (2). Many viruses, including RSV through its two nonstructural proteins (NS1 and NS2), therefore actively suppress apoptosis to promote viral replication (3). Necroptosis is another form of programmed cell death that, unlike apoptosis, leads to release of cellular contents into the extracellular environment, promoting inflammation (4). Necroptosis is caspase independent and occurs via receptor-mediated activation of RIPK1 (receptor-interacting serine/threonine-protein kinase 1) and RIPK3 and formation of the necrosome complex. Ensuing oligomerization of MLKL (mixed lineage kinase domain-like pseudokinase) disrupts the cell membrane, allowing release of damage-associated molecular patterns such as HMGB1 (high mobility group box 1) (5) (Figure 1). The consensus therefore is that necroptosis is a “fail-safe” form of cell death, limiting viral spread while alerting the immune system to danger. Indeed, as with apoptosis, many viruses have evolved strategies to limit necroptosis to promote replication (6).

**Figure 1.**
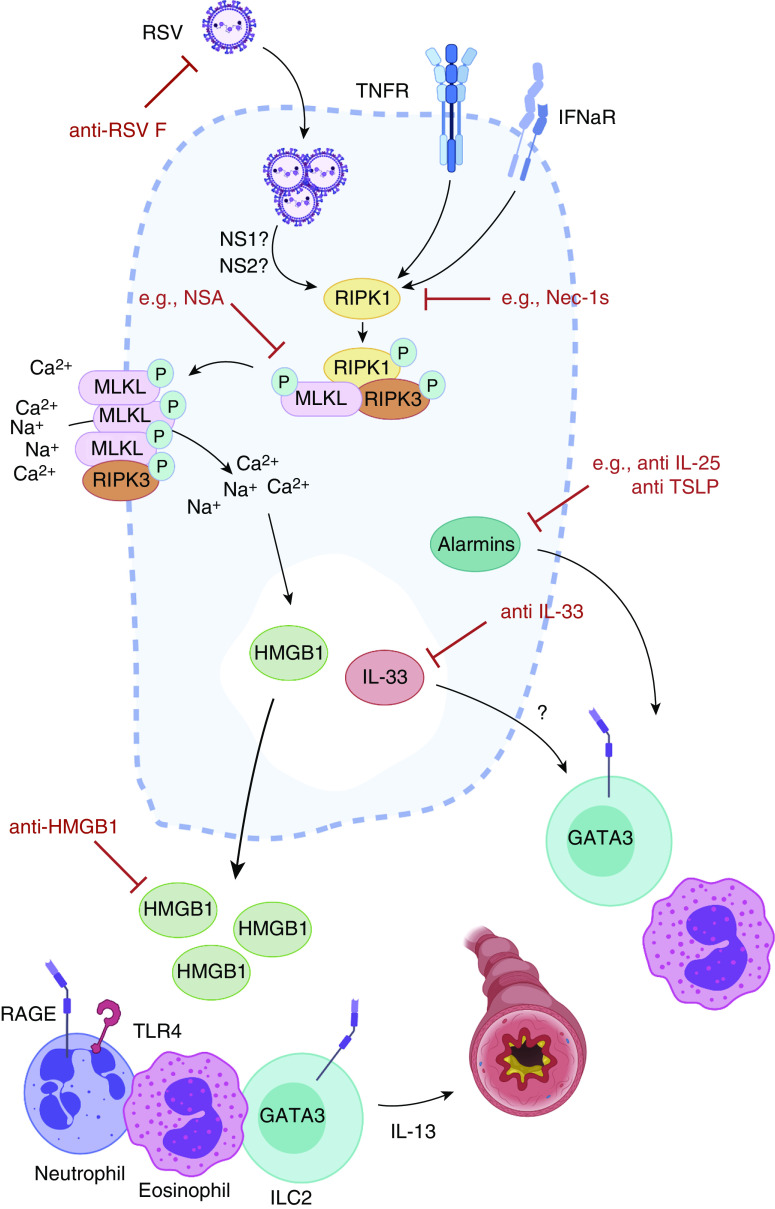
Can respiratory syncytial virus (RSV)-dependent necroptosis be targeted for therapeutic benefit? Simpson and colleagues demonstrate that RSV infection readily induces necroptosis in RSV-infected airway epithelial cells. The subsequent release of HMGB1 into the extracellular space results in the recruitment of proinflammatory and type 2–skewed immune responses, exacerbated disease, and an ensuing heightened susceptibility to asthma. This newly described pathway could potentially be targeted at multiple levels for therapeutic intervention during RSV bronchiolitis, as depicted by the red inhibitory arrows. GATA3 = GATA3 binding protein; HMGB1 = high mobility group box 1; IFNaR = IFN-α receptor; ILC2 = group 2 innate lymphoid cell; MLKL = mixed lineage kinase domain-like pseudokinase; Nec1s = 7-Cl-O-Necrostatin 1; NS1 = nonstructural protein 1; NS2 = nonstructural protein 2; NSA = necrosulfonamide; P = phosphorylated; RAGE = receptor for advance glycation end products; RIPK1 = receptor-interacting serine/threonine-protein kinase 1; RIPK3 = receptor-interacting serine/threonine-protein kinase 3; TLR4 = Toll-like receptor 4; TNFR = tumor necrosis factor receptor; TSLP = thymic stromal lymphopoietin.

In this issue of the *Journal* (pp. 1358–1371), Simpson and colleagues (7) challenge this classical dogma, arguing that in the context of RSV infection, necroptosis is detrimental to viral clearance and accentuates immunopathology and ensuing propensity to develop asthma. The authors show that HMGB1 is elevated in the nasopharynx of children specifically infected with RSV compared with those infected with other viruses. Subsequently, *in vitro* infection of healthy infant–derived AECs elicited necroptosis-dependent HMGB1 translocation and release that was associated with a reduction in viral titers. In a series of complementary studies, the authors show that pneumovirus (mPV; murine RSV ortholog) infection of neonatal mice also results in epithelial necroptosis and HMGB1 release, especially in mice deficient for the central IFN-stimulated gene *IRF7* (IFN regulatory factor 7). Subsequent pharmacological inhibition or genetic ablation of necroptosis markedly attenuated AEC sloughing and HMGB1 release while importantly reducing viral titers, neutrophilic and type 2 inflammation, and airway remodeling. Furthermore, pharmacological inhibition of necroptosis during primary mPV infection protected mice from subsequent development of experimental asthma.

This study importantly identifies necroptosis as a prominent cell death pathway initiated by RSV infection and delineates its downstream consequences in terms of immunity and pathology. Inevitably, however, unanswered questions persist that should remain the focus of future studies. The underlying mechanism by which RSV elicits AEC necroptosis, particularly the role of RSV proteins, remains unexplored. Findings derived from the mPV model suggest that augmented viral titers are associated with heightened necroptosis, and the authors understandably speculate a role for viral TLRs (Toll-like receptors) or inflammatory cytokines in necroptosis induction. Although both influenza A virus and RSV NS proteins function to suppress AEC apoptosis, it is intriguing that influenza A virus NS1 has also been shown to operate to induce necroptosis (8). Do RSV NS proteins therefore display dual roles in defining the apoptosis–necroptosis balance? If RSV-induced necroptosis is more prevalent in the context of impaired antiviral innate immunity, then host determinant factors are likely critical in defining the scale of necroptosis and ensuing adverse sequelae. Polymorphisms in key IFN and innate immune genes are the most significantly associated with compromised viral control and severe RSV bronchiolitis (9), genes that are also heavily linked to the development of asthma. Could impaired antiviral responses of asthmatic AECs (10) potentiate necroptosis and also be of relevance to virus-driven asthma exacerbations with associated augmentation of neutrophilia and type 2 inflammation?

The authors convincingly demonstrated the marked capacity of RSV/mPV to induce AEC necroptosis/HMGB1 release and the profound benefits of inhibiting necroptosis. It will be important, however, to delineate if and how HMGB1 is responsible for driving all purported downstream effects of necroptosis. HMGB1 binds a range of receptors, including RAGE (receptor for advance glycation end products) and TLR4, to promote the activation and recruitment of innate immune cells, including macrophages and neutrophils (5). Moreover, HMGB1 can act via RAGE to induce pulmonary group 2 innate lymphoid cell (ILC2) accumulation by promoting these cells’ proliferation and survival (11). Thus, it is easy to rationalize the necroptosis dependency of neutrophilic, ILC2, and eosinophilic inflammation and ensuing airway remodeling after mPV infection. Virally induced necroptosis also likely facilitates the release of prototypical alarmins associated with induction of type 2 responses, such as IL-33, IL-25, and TSLP (thymic stromal lymphopoietin) (12), and thus it would be intriguing to evaluate their significance within this pathway. It is also clear that necroptosis during RSV infection can play a role beyond the epithelium, because RSV-exposed neutrophils (potentially recruited secondarily to HMGB1) can undergo necroptosis and ensuing NETosis (13), with neutrophil extracellular traps also having been demonstrated to be potentiators of type 2–driven immunopathology (14). It also remains to be determined how inhibition of necroptosis improves viral clearance independent of an augmented IFN response. Moreover, given that necroptosis has conversely been demonstrated to be beneficial to control of numerous other viruses (6), it would be important to ascertain what defines this virus-specific role for necroptosis in host immunity.

In the future, it will be important to extend clinical aspects of this study to unequivocally demonstrate that severe RSV-driven bronchiolitis in infants directly correlates with evidence of heightened AEC necroptosis. Although inherent challenges exist in elucidating the cause and effect in such a scenario, initial evidence suggests that local HMGB1 levels may correlate with disease severity (15), but its association with viral titers and the relevant inflammatory markers is unclear. Moreover, although clearly challenging, it would be intriguing to delineate whether evidence of a robust necroptotic response during primary RSV infection associates with a greater risk of developing asthma later in life. Given the findings of the study by Simpson and colleagues (7), there is a clear opportunity to target necroptosis at various levels for therapeutic intervention during severe RSV bronchiolitis (Figure 1). The added specificity of targeting downstream mediators such as HMGB1 would seem preferable if, as discussed above, it can be shown to be the instigator of all adverse sequelae attributed to RSV-driven AEC necroptosis. Given the aforementioned conflicting beneficial roles attributed to necroptosis for distinct viral infections, it would, of course, be prudent to validate that such strategies do not render children more susceptible to other infections.

## Supplementary Material

Supplements

Author disclosures
