# Supplementary material for: A Not-So-Good Way to Die? Respiratory Syncytial Virus–induced Necroptotic Cell Death Promotes Inflammation and Type 2–mediated Pathology
Source: Am J Respir Crit Care Med. 2020 Jun 1;201(11):1321–3. doi: 10.1164/rccm.202003-0533ED (PMC7258638; doi:10.1164/rccm.202003-0533ED)
Supplement: Supplements [file rccm.202003-0533ED.html]

A Not-So-Good Way to Die? Respiratory Syncytial Virusâ€“induced Necroptotic Cell Death Promotes Inflammation and Type 2â€“mediated Pathology | American Journal of Respiratory and Critical Care Medicine

- disclosures.pdf (153 KB)
